# A comprehensive mapping of the current capacity for human nutrition training in Cameroon

**DOI:** 10.3402/gha.v9.29548

**Published:** 2016-01-25

**Authors:** Roger Sodjinou, Ines Lezama, Marie-Louise Asse, Georges Okala, William K. Bosu, Nadia Fanou, Ludvine Mbala, Noel Marie Zagre, Félicité Tchibindat

**Affiliations:** 1 UNICEF Regional Office for West and Central Africa, Dakar, Senegal; 2 West Africa Health Organization (WAHO), Bobo-Dioulasso, Burkina Faso; 3 UNICEF Cameroon, Yaoundé, Cameroon; 4 Ministry of Higher Education, Yaoundé, Cameroon; 5 Ministry of Public Health, Yaoundé, Cameroon; 6 Department of Nutrition and Food Science, University of Abomey-Calavi, Abomey-Calavi, Benin

**Keywords:** nutrition, training, public health nutrition, capacity, Cameroon

## Abstract

**Background:**

There is consensus among stakeholders in Cameroon on the need to develop and strengthen human resource capacity for nutrition. This study was conducted to provide a comprehensive mapping of the current capacity for tertiary-level human nutrition training in Cameroon.

**Design:**

Participating institutions included university-level institutions offering dedicated nutrition degree programs or other programs in which nutrition courses were taught. A semi-structured questionnaire administered during in-person interviews was used to collect data on existing programs and content of training curricula. Nutrition curricula were reviewed against the following criteria: intended objectives, coverage of nutrition topics, and teaching methods.

**Results:**

In total, five nutrition degree programs (four undergraduate programs and one master's program) were identified. Three additional programs were about to be launched at the time of data collection. We did not find any doctorate degree programs in nutrition. All the undergraduate programs only had little focus on public health nutrition whereas the master's program in our sample offered a good coverage of all dimensions of human nutrition including basic and applied nutrition. The predominant teaching method was didactic lecture in all the programs. We did not find any formal documentation outlining the competencies that students were expected to gain upon completion of these programs. Nutrition courses in agricultural and health schools were limited in terms of contact hours and scope. Public health nutrition was not covered in any of the health professional schools surveyed. We found no institution offering in-service nutrition training at the time of the study.

**Conclusions:**

Based on our findings, we recommend that nutrition training programs in Cameroon be redesigned to make them more responsive to the public health needs of the country.

## Introduction

**Fig. 1:**
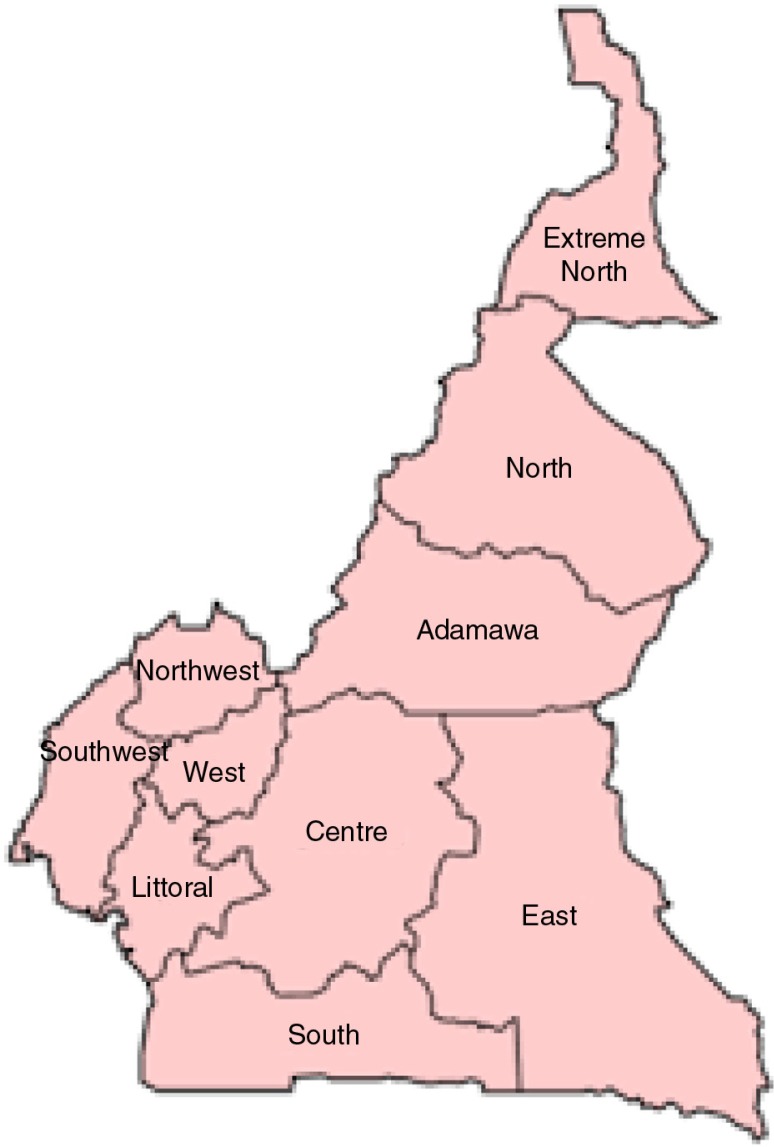
Administrative regions of Cameroon.

Cameroon is a Central African country with a population of approximately 23 million (1). It is divided into 10 administrative regions (Fig. 1). Life expectancy at birth is 55 years. Cameroon, with a gross national income per capita of US$1,350, is classified as a low-to-middle income country by the World Bank (2).

Important efforts have been made over the past years to raise the profile of nutrition to a high priority in Cameroon. The country joined the Scaling Up Nutrition (SUN) movement in March 2013 (3). The SUN movement supports national governments to work through multisectoral, multistakeholder platforms; align programs toward common objectives and agreed frameworks for results; and mobilize domestic and external resources to foster these efforts (3). Cameroon's membership of the SUN movement has galvanized political commitment to improve governance and accelerate progress in nutrition. Efforts are underway to develop a coherent multisectoral policy for nutrition, align nutrition programs toward a common results framework, and improve clarity over investments in nutrition and budgeting (3). The Government of Cameroon is also taking steps to create a multisectoral platform for nutrition that will be managed at a higher level in the political hierarchy. A network of parliamentarians for the fight against undernutrition is very active in the country. Much work is being done to leverage additional resources for nutrition through innovative financing mechanisms such as engagement with the private sector (3).

Despite these positive developments, malnutrition remains a serious problem in Cameroon. The prevalence of child stunting is high (32.6%), with nearly 1 million under-five children being affected (4). In addition, 5.6% of children are wasted (4). Micronutrient deficiencies are also common in Cameroon. In a nationally representative survey, the prevalence of iron-deficiency anemia ranged from 12.0 to 47.4% among children and from 9.0 to 19.4% among women (5). The prevalence of vitamin A deficiency among under-five children is 39%, whereas it is 18% among pregnant women (6). At the same time, the country is experiencing a nutrition transition fueled by a rapid urbanization and changes in diet and lifestyle patterns; with an increase in overnutrition and non-communicable diseases (NCDs). Nearly 4% of under-five children in Cameroon are overweight (4). In addition, the proportion of adult women who were overweight or obese increased from 20.7% in 1998 to 31.3% in 2011 (7). The prevalence of hypertension increased 2.3–3.2 times in rural Cameroon and 1.6–1.9 times in urban Cameroon over a 10-year period (8).

Cameroon faces an acute shortage of human resources in the health sector and in their equitable distribution across the country (9). The lack of human resources in the health sector in Cameroon is likely one of the factors hindering Cameroon's progress in nutrition. Although there is a strong consensus among stakeholders on the urgent need to develop capacity for nutrition at all levels, investments in capacity development initiatives in the country have so far been limited. The recent Lancet series (10) and other studies (11, 12) emphasize the need to strengthen institutional, organizational, and human capacity to scale up nutrition interventions and accelerate progress in nutrition. Failure to develop the needed capacity for action in nutrition will ultimately slow down progress in nutrition in the country. A preliminary step in capacity development is to assess the capacity assets and gaps that exist at individual, organizational, and systemic levels as done recently in West Africa (13–15).

Developing a workforce capacity will not address all capacity gaps in a sustainable manner because capacity development goes beyond workforce development and the training of individuals (15, 16). As described by Potter and Brough (16), it covers a set of three separate but interrelated elements, which are individual capacity (skills and tools), organizational capacity (staff and infrastructures), and systemic capacity (systems, structures, and roles). This paper assesses the current capacity for human nutrition training in Cameroon, in terms of the nutrition programs offered by educational institutions (organizational level) and the skills and competencies acquired by students in the different programs (individual level). The existing nutrition capacity assets and gaps at the systemic level in Cameroon will be addressed in a separate paper.

The paper also highlights existing gaps and challenges in current approaches to nutrition training in the country and proposes practical solutions to address them. The study was commissioned by the Government of Cameroon (Ministry of Higher Education and Ministry of Public Health), with the support of UNICEF, Helen Keller International, and other partners.

## Methods

### Participating institutions

There were two levels of selection. First, we screened a set of institutions based on a preliminary list that was provided by the Ministry of Public Health, the Ministry of Higher Education, and key in-country informants. These institutions were contacted by telephone or email to check their eligibility. Then, we enrolled those institutions found to run dedicated nutrition degree programs or those offering other programs in which nutrition courses were taught (Table 1).

**Table 1:** Number of participating institutions and training programs assessed

Region	City	No. of participating institutions	Number of training programs assessed	No. of institutions offering dedicated nutrition courses		No. of institutions offering nutrition integrated into other courses

				Undergraduate	Masters	
Adamawa	Ngaoundere	3	6	–	1	2
Northwest	Bamenda	5	9	1	1	3
Far North	Maroua	2	5	1	1	0
West	Dschang	2	4	–	–	2
Center	Yaoundé	7	16	1	–	6
Littoral	Douala	9	18		–	9
Southwest	Buea	2	3	1	–	1
Total		30	61	4	3	23

An official letter was sent to all targeted institutions by their respective line ministries to explain the objectives of the assessment and request their participation. In-person meetings were then scheduled with the representatives of the institutions to gather information about existing nutrition programs or courses. During the meeting, the objectives of the assessment were further explained to the respondents. Interviews were conducted either in French or English.

### Data collection

There were two rounds of data collection for this assessment. The first round, which took place in 2013, was conducted in four regions (Center, West, Southwest, and the Littoral). The second round, which took place in 2014, also covered the West region and three other regions (Far North, Adamawa, and Northwest). In total, seven of the ten regions of the country were covered (Fig. 1).

The data collection method used was similar to the one used to assess the capacity for nutrition training in West Africa as described elsewhere (13–15). Data were collected using a semi-structured open-ended questionnaire administered by four experienced interviewers during face-to-face interviews. The questionnaires were completed manually by the interviewers in the presence of the representatives of the institutions (or the persons designated by them). Information was gathered on existing programs and content of training curricula, school ownership, and institutional collaborations. In agricultural and health professional schools, data were also collected on the total number of hours devoted to nutrition across the curriculum, the content and status of nutrition courses, the distribution of nutrition instruction throughout the curriculum, the periods of the curriculum in which nutrition was taught, and the prevailing teaching methods.

### Data analysis

Existing academic nutrition programs in the country were reviewed against the following criteria:

- Outline of the objectives of the programs (skills and knowledge to be taught).
- Coverage of nutrition topics in the curriculum. Three main categories were defined to characterize the areas of nutrition emphasized in the curriculum: basic nutrition, clinical nutrition, and public health nutrition. Basic nutrition was defined as the application of nutrition principles at cellular or organ level and clinical nutrition as the application of nutrition principles at the individual level in clinical settings. Public health nutrition was defined in the context of this study as the use of health promotion approaches and the application of public health principles to nutrition at the population level to prevent nutrition-related diseases, promote health, and improve overall health outcomes (17, 18).
- Training design and teaching method. The prevailing teaching methods were categorized as didactic method (focus on the acquisition of knowledge), problem-based learning (focus on the acquisition of knowledge, abilities, and skills), and a holistic, integrated approach (focus on the acquisition of knowledge, skills, and competencies) (14).

## Results

### Undergraduate programs in nutrition

In total, four undergraduate programs in nutrition were available in three regions in Cameroon at the time of data collection. These programs were found in Bamenda, Buea, and Yaoundé (Table 2). The University of Maroua was about to launch a bachelor's program in nutrition at the time of the study.

**Table 2:** List of universities that provide undergraduate training programs in nutrition in Cameroon

City	Institution	Ownership	Faculty	Degree awarded	Program duration (months)	Year program inception	Language of instruction	Primary teaching method
Yaoundé	Institut Supérieur des Sciences Biologiques et Appliquées (ISSBA)	Private	–	Higher Diploma in Dietetics	24	2011	French	Didactic lecture
Buea	St. Francis Higher Institute of Nursing and Midwifery	Private	–	Bachelor in Nutrition and Dietetics	36	2013	English	Didactic lecture
				Higher National Diploma in Nutrition and Dietetics	24	2013	English	Didactic lecture
Bamenda	University of Bamenda	Public	College of Technology	Bachelor of Technology-Nutrition	60	2013	English	Didactic lecture
Maroua	University of Maroua	Public	Faculty of Science	Professional bachelor's degree in Health and Nutrition	36	The program was about to be launched	French and English	Didactic lecture

All the programs were established during the 5 years preceding the survey. The program in Bamenda was run by a government-supported institution, whereas the programs in Yaoundé and Buea were run by private institutions. None of the programs was involved in international collaboration for nutrition training or research.

The Yaoundé and Buea programs award a higher national diploma (HND). The Buea and Bamenda programs award bachelor-level degrees. The language of instruction in Buea and Bamenda is English whereas that in Yaoundé is French.

The admission requirements for all the undergraduate programs are a technical or general college education with a science background or any other equivalent qualifications as accepted by the Ministry of Higher Education of Cameroon. The full-time duration of the bachelor degree program in Buea is 3 years whereas that of Yaoundé and Bamenda is 24 months and 60 months, respectively. The HND in Nutrition and Dietetics offered in Buea is of 24 month-duration.

The teaching format for all the programs was didactic with little practical training. The program in Bamenda was heavy on basic nutrition and food science, although it included some courses on community nutrition and clinical nutrition. In contrast, the curricula of the Buea and Yaoundé programs emphasized dietetics and applied nutrition. The nutrition courses offered were mainly on food, nutrition, and dietary planning. None of the programs emphasized public health nutrition, or the ongoing nutrition transition in the country, or the attendant rise in NCDs as a key area in their curricula. Furthermore, none of them adequately covered the national protocol for the management of acute malnutrition. The detailed curricula are available upon request.

We did not find any formal documentation outlining the competencies that students were expected to gain upon completion of these programs.

### Graduate programs in nutrition

The University of Ngaoundere was the only institution offering a graduate program in nutrition in Cameroon at the time of data collection (Table 3). The 2-year program was established in 2010. Courses are taught in both French and English. The admission requirement is a bachelor of science (BSc) degree in biological sciences, nutrition, biochemistry or any other related subjects. The teaching format was mainly didactic, and the program was by coursework. In order to graduate, students were required to undergo a 6-month internship in a professional setting and write a dissertation.

**Table 3:** List of universities that provide master's training programs in nutrition in Cameroon

City	Institution	Type of institution	Faculty/center	Degree awarded	Program duration (months)	Year program inception	Language of instruction	Student intake (2014)	Primary teaching method
Ngaoundere	University of Ngaoundere	Public	Ecole Nationale Supérieure des Sciences Agro-Industrielles (ENSAI)	Professional master's degree in Applied Nutrition	24	2010	French and English	30	Didactic lecture
Maroua	University of Maroua	Public	Faculty of Science	Master's in Food and Nutritional Biochemistry	24	The two programs was about to be launched	French and English	–	Didactic lecture
Bamenda	University of Bamenda	Public	College of Technology	Master's in Nutrition	24	The program was about to be launched	English	–	Didactic lecture

The curriculum offered a good coverage of the key dimensions of human nutrition (Table 4). The curriculum focused on some of the aspects of basic nutrition (nutrition in the life span, nutrition physiology and biochemistry, nutritional assessment, food consumption, regulation of nutrient metabolism, maternal and child nutrition, etc.). It also provided students with the opportunity to gain insights into some of the major aspects of applied nutrition and dietetics (menu planning; diet therapy; cooking techniques; therapeutic nutrition; food consumption studies; applied nutrition). Public health nutrition was also covered in the training curriculum (60 h in total). As with the undergraduate programs, we did not find any formal documentation outlining the competencies that students were expected to gain throughout the program.

**Table 4:** Detailed curriculum for the professional master's degree program in applied nutrition – ENSAI Ngaoundere

Semesters	Curriculum content (total number of hours)
Semester 1	Biostatistics and introduction to research methods (30 h)
	Food safety and hygiene legislation (30 h)
	Food Science (45 h)
	General microbiology and Food microbiology (45 h)
	Human anatomy and physiology (45 h)
	Nutrition in the life span (60 h)
	Food biochemistry and toxicology (45 h)
	Food and Nutrition (45 h)
	Scientific English and communication techniques (45 h)
	Nutritional physiology and biochemistry (60 h)
Semester 2	Nutrition and infection (45 h)
	Human physiopathology (45 h)
	Menu planning (30 h)
	Evaluation of nutritional status and food consumption studies (45 h)
	Introduction to diet therapy (45 h)
	Cooking techniques (30 h)
	Public health nutrition (60 h)
	Food Science 2 (45 h)
	Nutritional Epidemiology (45 h)
	Applied nutrition internship (3 months)
Semester 3	Advanced macronutrient metabolism (45 h)
	Sensory evaluation (30 h)
	Food meal management and service (30 h)
	Nutrition intervention-Development (30 h)
	Current topics in human nutrition and dietetics
	Food and drug interaction (15 h)
	Therapeutic nutrition (45 h)
	Community and international nutrition (45 h)
	Herbs, functional foods and phytochemicals (30 h)
	Nutrition and behavior/psychology (30 h)
	Nutrition education (45 h)
	Research methods in human nutrition (30 h)
Semester 4	Food economics (45 h)
	Fundamentals of sports nutrition (30 h)
	Clinical nutrition (45 h)
	Maternal and child nutrition (30 h)
	Internship and dissertation (6 months)

For each course, 70% of the total instruction time was devoted to didactic learning and 30% to hands-on practicum.

The University of Maroua and the University of Bamenda, two state-owned universities, were also about to launch master's programs related to nutrition at the time of data collection (Table 3).

Although we did not find any doctorate degree programs in nutrition in Cameroon, we noted that there were some Ph.D. holders who had obtained their degrees in nutrition abroad.

### Nutrition training in nursing, midwifery, and medical schools

All the nursing and midwifery schools surveyed used a standard nutrition curriculum that was designed by the Ministry of Public Health of Cameroon (Table 5). Nutrition instruction was given mainly during the first year of the nursing and midwifery programs, whereas it was given throughout the duration of the medical school program, especially during clinical years (Table 5). The total amount of time devoted to nutrition was 37 h in the nursing program, 20 h in the midwifery program, and 28 h in the medical program.

**Table 5:** Standard nutrition curricula in health professional schools in Cameroon

Program	Nutrition course taught	Distribution of nutrition instruction throughout the curriculum	Teaching format	Status of nutrition courses	Year in the curriculum	Hours of contact for nutrition courses			

						Total	Didactic courses	Hands-on training	Other
Nursing program	Food and food groups Nutritional requirements Nutritional disorders	Stand-alone course	Didactic	Required	First cycle (first year)	37	30	5	2
Midwifery program	Infant feeding Maternal feeding Weaning foods	Stand-alone course	Didactic	Required	First cycle (first year)	20	12	6	2
Medical program^a^	Breastfeeding and dietary diversification	Stand-alone course	Didactic	Required	Second cycle (third year)	4	4	0	0
	Clinical nutrition and infant feeding	Integrated with other courses	Integrated system-based	Required	Second cycle (fourth year)	20	17	1	2
	Acute malnutrition in children	Stand-alone course	Didactic	Required	Second cycle (fifth year)	4	0	0	0

a Some nutrition topics were also embedded in other clinical courses in medical schools. The topics are not presented here.

Only the nutrition content of the curriculum of the Faculty of Medicine of Douala is presented here. There are at least 10 faculties offering medical training in the country (9).

Unlike medical schools where nutrition was embedded in other clinical courses, nutrition instruction was delivered mainly as a stand-alone course in nursing and midwifery schools. As with the undergraduate nutrition degree programs, the teaching method for nutrition training in the health professional institutions was mainly didactic, with little opportunity given to students to apply the basic principles of human nutrition to public health practice.

The training content in nursing and midwifery schools was heavily oriented toward basic nutrition. The nursing programs included three courses on food and food groups, nutritional requirements, and nutritional disorders (Table 5). Some of the foods included in the curriculum were not aligned with the eating habits of the population. The midwifery programs included infant feeding, maternal diet, and weaning foods. The medical schools’ programs included breastfeeding, clinical nutrition, infant feeding, and acute malnutrition in children (Table 5). Public health nutrition was not covered in any of these curricula.

### Nutrition training in other university-level institutions

Several university-level institutions in Cameroon offered nutrition courses as part of the curricula for other programs (Table 6). These included University of Yaoundé 1 (Faculty of Science), University of Buea (Faculty of Science), University of Douala (Faculty of Science; ENSET), University of Maroua (Faculty of Science; Higher Institute of Sahel), University of Ngaoundere (Faculty of Science; ENSAI; Higher Institute of Technology), and University of Bamenda (ENSET; Bamenda University of Science and Technology). In general, these courses were mostly in basic nutrition and food science, particularly nutritional biochemistry. They were limited both in terms of coverage and duration. However, the programs at University of Bamenda offered good coverage of some of the basic principles of human nutrition (Table 6).

**Table 6:** List of selected universities that provide nutrition courses as part of their training curricula in Cameroon

City	Institution	Ownership	Faculty/Center	Degree awarded	Nutrition courses taught	Number of hours devoted to nutrition
Dschang	University of Dschang	Public	Faculty of Science	Bachelor's in Biochemistry Bachelor's in Health Sciences	Human nutrition	60
Buea	University of Buea	Public	Faculty of Science	Bachelor's in Biochemistry	Nutrition biochemistry	60
Yaounde	University of Yaoundé 1	Public	Faculty of Science	Master's in Biochemistry (alternative sciences and nutrition)	Nutrition biochemistry	60
	Ecole des Sciences de la Santé	Private	Centre Supérieur des Sciences de la Santé	Master's in Public Health	Nutritional epidemiology	20
				Master's in Reproductive Health	Young child feeding	16
				Master's in Nursing Sciences	Nutritional epidemiology	20
				Bachelor's in Nursing Sciences	Nutrition and nutritional disorders	25
					Nutrition and dietetics	20
Douala	University of Douala	Public	Faculty of Science	Master's in Biochemistry	Nutritional biochemistry	44
			Higher Technical Teachers Training College (ENSET)	Diplôme de l'Ecole Normale Supérieure de l'Enseignement Technique	Nutrition 3	45
					Nutrition and culinary arts	45
					Nutrition and health	45
					Nutrition and food environment	45
	Institut Universitaire et Stratégies de l'Estuaire	Private	Institut Universitaire et Stratégies de l'Estuaire	Master's in Medical Biochemistry	Human nutrition	60
	Higher Institute for Nursing	Private	Higher Institute for Nursing	Diplôme Supérieur d'Etudes Professionnelles en Soins Infirmiers	Nutrition	37
Maroua	University of Maroua	Public	Institut Supérieur du Sahel (ISS)	Bachelor's level 3 (agrifood)	Introduction to human nutrition	45
				Master's level 1 (agrifood)	Advanced human nutrition	45
			Faculty of Science	Master's in Food and Nutritional Biochemistry	The program was being validated at the time of data collection	–
Ngaoundere	University of Ngaoundere	Public	Faculty of Science	Bachelor's inBiomedical and Medico-Sanitary	Dermatology-nutrition	60
				Master's in Biomedical and Medico-Sanitary Science	Homeostasis and nutrition	60
			Institut Universitaire de Technologie (IUT)	Diplome Universitaire de Technologie (DUT) and Bachelor's in Food Industries and Biotechnology	Nutritional biochemistry	65
			Ecole Nationale Supérieure des Sciences Agro-Industrielles (ENSAI)	Engineer – Agricultural and Agri-Business Industries	Human nutrition	45
				Master's in Science and Technology (with specialization in Food Security and Nutrition)	Nutritional biochemistry	30
					The state of food security and nutrition in the world (basics and advanced)	75
					Nutritional intervention and development	30
					Nutrition education	30
Bamenda	University of Bamenda	Public	Higher Technical Teachers Training College (ENSET)	Food Science and Technology Program/Home Economics Program	Introduction to nutrition	30
					Human nutrition	60
					Nutritional assessment	45
					Nutrition education and counselling	90
					Disease and therapeutic nutrition	120
					Advanced nutrition	30
					Nutrition and counselling	45
			Bamenda University Science and Technology (BUST)	Bachelor of Technology (Food Science and Nutrition; Food Science and Technology; Home Economics)	Fundamental of health and nutrition	67
					Nutrition and body weight	69
					Metabolism and energy balance	60
					Nutrition for pregnancy, childhood and adolescence	69
					Nutrition for adults and elderly	60
					Nutrition and physical fitness	68
					Special teaching methods in food science and nutrition	48
					Research seminar in food science and nutrition	36
					Research project in food science and nutrition	37

Nutrition was also taught in some programs offering degrees in agriculture and related areas. These included master's programs in animal production (University of Dschang; University of Maroua), Master's in Crop Production (University of Dschang), and an Agricultural Engineering degree (University of Bamenda).

### Short-term professional training in nutrition

There were limited opportunities for continuing professional development in nutrition. There were no short-term nutrition training programs available in the country at the time of the study.

## Discussion

In this study, we provided a detailed assessment of the current academic nutrition programs in Cameroon. We found that university-level nutrition training was not common. We identified only eight nutrition degree programs in the country, five of which were less than age 5 and three were about to start. Contrary to study findings in West Africa (13), we did not find any doctorate degree programs in nutrition or any institution offering in-service nutrition training at the time of the study. With the planned start of additional nutrition degree programs and the active involvement of the private sector, there are good prospects for improving nutrition capacity in Cameroon, at least for strengthening the human resource base for nutrition. However, the development of new degree programs needs to be accompanied by a comprehensive national strategy that gives clear indications about what is needed in terms of human capacity, financial resources, and logistical support.

The study revealed several weaknesses in the existing nutrition training programs in terms of the opportunities to acquire competencies for nutrition career practice, teaching methodology, the scope of topics covered, alignment with national priorities, and engagement in international collaboration. Overall, the findings of this study are consistent with recent observations about nutrition training programs in West Africa (13), and in South Asia (19, 20). The curriculum at the University of Ngaoundere covered most of the essential elements of human nutrition, and so could serve as a model for the development of a harmonized curriculum across the country, at least for master's programs in nutrition.

We did not find any formal documentation outlining the competencies that students were expected to gain in any program. The need for competency-based nutrition training in Africa has been stressed by many experts (21, 22). To ensure that programs in Cameroon respond to national nutrition priorities, nutrition service providers need to know how to manage acute malnutrition using the national protocol, promote appropriate infant and young child feeding practices, and prevent micronutrient deficiencies and diet-related chronic diseases through the application of public health principles (14). Apart from technical skills, they should also be able to work cross-sectorally and exhibit strong leadership, advocacy, and communication skills (13). A skilled nutrition workforce is crucial for the implementation of effective nutrition policies and actions (23, 24). Training institutions should work together to come up with the key competencies that need to be acquired at each level of nutrition training in Cameroon. This will also require the active collaboration between the training institutions and the employing agencies. This would provide the basis for the revision of the existing training curricula so that they are better aligned with national nutrition priorities and include practical training.

We also found that public health nutrition was only marginally covered in both dedicated nutrition degree programs and in health professional schools. The need to focus on public health nutrition to improve nutrition outcomes in a sustainable manner is of paramount importance. A lack of trained professionals in public health nutrition is one the factors hindering the implementation of nutrition interventions (19, 24).

Unlike the situation in West Africa, we found no short-term professional nutrition courses in Cameroon. Continuous professional education programs or in-service training are important because they can provide the opportunities for updating knowledge, skills, and competencies in nutrition. However, this approach will only be effective if the basic training of beneficiaries is adequate and sufficient time and resources are allocated to it. Although the capacity for supervisory training, mentoring, and on-the-job nutrition training in Cameroon was beyond the scope of this study, there is also a need to document and improve current practices because most organizations in the country use these strategies to build the capacity of their staff on nutrition.

Even more importantly, individual capacity development efforts need to go beyond training staff from the health sector. Non-medical staff, especially those from agriculture and WASH (water, sanitation and hygiene) sectors, need to be equipped with adequate knowledge on nutrition to help accelerate the delivery of nutrition-sensitive interventions. Training curricula in agriculture schools also need to be reviewed to strengthen the linkages between agriculture and nutrition and provide a platform for scaling up nutrition-sensitive interventions (25).

Our results also revealed that collaboration and networking between training institutions in Cameroon were not well established and need to be strengthened. Enabling closer ties between training institutions inside the country could foster the mobility of students and faculty members across the country (13). However, it is important to clearly define the areas of collaboration so that there are mutual benefits for all institutions involved.

There are several limitations for this study that should be noted. First, our inventory may not have been exhaustive as we covered only seven out of ten regions. However, these were the regions that contained the largest universities, where tertiary-level training was likely to be located. In spite of our best efforts to provide a comprehensive assessment of the current academic nutrition offerings in Cameroon, some programs may have been omitted. However, we believe any gaps are unlikely to significantly affect our findings. Secondly, we do not sufficiently discuss the steps that need to be taken to improve the quality of nutrition training in Cameroon. We are planning to conduct a workshop in Cameroon during which the key results of this study will be discussed with the Government of Cameroon and all stakeholders. This will ultimately pave the way for the development of a consensual action plan that will help to upgrade existing programs and develop new programs that are of high quality.

## Conclusions

This study is a first step toward the strengthening of human capacity for nutrition in Cameroon. Our findings highlight the need for a shift in paradigm for nutrition training in Cameroon to be more responsive to the public health needs in the country. Nutrition training programs could be improved by making them more comprehensive in the coverage of all dimensions of human nutrition (basic nutrition, clinical nutrition, and public health nutrition), designing more practical-based training, emphasizing competencies, and harmonizing nutrition curricula to ensure that a minimum set of competencies are present in the workforce. It is also vital to enhance in-service nutrition training for health professionals in the country and support the establishment of new nutrition degree programs to enable an expansion of a nutrition workforce across the country. The Government of Cameroon, through the Ministry of Higher Education and the Ministry of Health, should also take steps to establish standards for systematically monitoring the quality of nutrition training programs to ensure that they are aligned to national nutrition priorities.
